# Supplementary material for: Identification of photoperiod-induced specific miRNAs in the adrenal glands of Sunite sheep (Ovis aries)
Source: Front Vet Sci. 2022 Jul 22;9:888207. doi: 10.3389/fvets.2022.888207 (PMC9354845; doi:10.3389/fvets.2022.888207)
Supplement: Supplementary file 1 [file Data_Sheet_1.ZIP › Supplementary file/miRDeep2 file.zip!/miRDeep2 file/Novel_Structure/SPLP42A3/Novel_177.pdf]

Provisional ID	:	NC_019470.2_23911
Score total	:	7.1
Score for star read(s)	:	3.9
Score for read counts	:	0.1
Score for mfe	:	1.4
Score for randfold	:	1.6
Score for cons. seed	:	
Total read count	:	12
Mature read count	:	7
Loop read count	:	0
Star read count	:	5

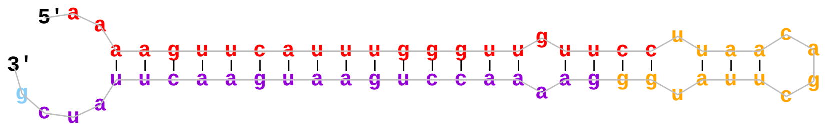

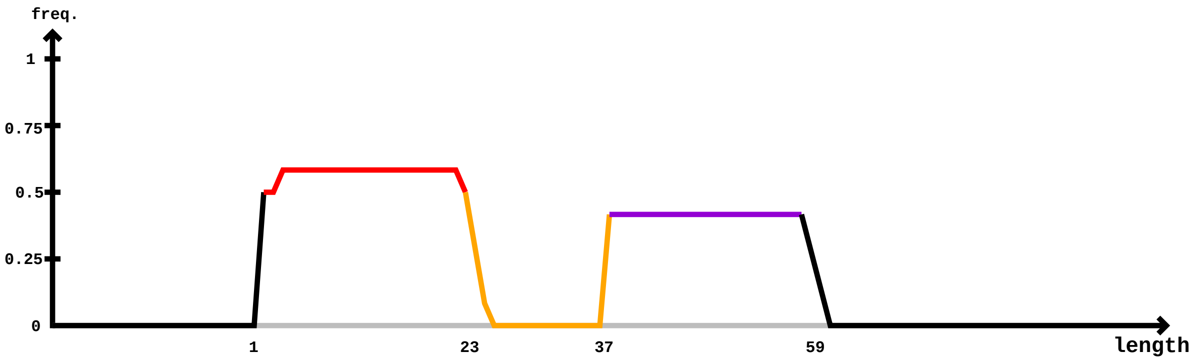

Mature Star

```
5'- uacuacuauuguguuggccaaaaaguucauuuggguuguuccuuaacagcuuauggggaaaaccugaaugaacuuaucggucaacccaaaacguuagagaaauguagagugacag   -3'    obs
    uacuacuauuguguuggccaaaaaguucauuuggguuguuccuuaacagcuuaugggaaaaccugaaugaacuuaucggucaacccaaaacguuagagaaauguagagugacag          exp
    ((((.((((((.(((((((...((((((((((((((.((((.(((....))).)))).)))))))))))))))...))))))).))..............))))))))....   reads    mm    sample
    ....................aaaaguucauuuggguuguuc.........................................................................   1        0     seq
    ....................aaaaguucauuuggguuguucc........................................................................   3        0     seq
    ....................aaaaguucauuuggguuguuccA.......................................................................   1        1     seq
    ....................aaaaguucauuuggguuguuccuu......................................................................   1        0     seq
    ......................aaguucauuuggguuUuucc........................................................................   1        1     seq
    ........................................................gaaaaccugaaugaacuuauc.....................................   3        0     seq
    ........................................................gaaaaccugaaugaacuuaucg....................................   2        0     seq
```
